# Subcutaneous Implantation of Open Microwell Islet Delivery Devices in Pigs

**DOI:** 10.1177/15533506241306491

**Published:** 2024-12-13

**Authors:** Maarten C. Tol, Rick H. W. de Vries, Marten A. Engelse, Françoise Carlotti, Aart A. van Apeldoorn, Eelco J. P. de Koning, Volkert A. L. Huurman

**Affiliations:** 1 Department of Internal Medicine, 4501 Leiden University Medical Center, Leiden, The Netherlands; 2 LUMC Transplant Center, 4501 Leiden University Medical Center, Leiden, The Netherlands; 3 Department of Cell Biology – Inspired Tissue Engineering (cBITE), 534871 MERLN Institute for Technology Inspired Regenerative Medicine, Maastricht University, Maastricht, The Netherlands; 4 Department of Surgery, 4501 Leiden University Medical Center, Leiden, The Netherlands

**Keywords:** biomedical engineering, tissue engineering, endocrine surgery

## Abstract

**Background:**

Intraportal pancreatic islet transplantation is a treatment option for patients with severe beta cell failure and unstable glycemic control. However, this procedure is associated with loss of beta cells after intrahepatic transplantation. Islet delivery devices (IDDs) implanted at extrahepatic sites may support engraftment and improve survival of pancreatic islets. We assessed the surgical feasibility, tolerability and safety of implantation of open microwell devices at subcutaneous sites with varying friction in pigs.

**Methods:**

Open, non-immunoisolating microwell islet delivery devices were made from polyvinylidene fluoride (PVDF). Empty (n = 26) and islet-seeded devices (n = 8) were implanted subcutaneously in 6 immunocompetent pigs in low-friction sites (abdomen and lateral hip) and high-friction sites (anterior neck) for 3 months. Retrieved grafts were analyzed histologically with haematoxylin and eosin, and Masson’s Trichrome staining.

**Results:**

Islet-seeding and transportation of IDDs was free from complications with minimal islet spillage. IDDs were implanted subcutaneously using standard surgical equipment, without complications during the surgeries. IDDs implanted in the neck and IDDs co-transplanted with human islets were expelled and retrieved after 10 days. Empty IDDs were removed after 3 months. The abdominal site showed reduced signs of inflammation as compared to the neck region, while similar tissue ingrowth and vascularization of devices were found in the two locations.

**Conclusions:**

Open microwell IDDs can safely be implanted with standard surgical equipment and successful islet-loading can be performed. Low-friction sites are preferable over high-friction sites for subcutaneous implantation in the porcine model since these lead to the least amount of foreign body reaction.

## Introduction

Allogeneic pancreatic islet transplantation ameliorates glycemic control and increases hypoglycemia awareness in patients with type 1 diabetes.^1^ Islets are infused into the portal vein and engraft in the liver. However, the liver is considered a suboptimal transplant site.^2^ Considerable islet mass is lost shortly after transplantation, partly attributed to the direct contact of islets with blood components leading to inflammatory and coagulation cascades, also known as the instant blood mediated immune response (IBMIR).^3,4^ Moreover, the liver is limited in the amount of tissue volume that can be transplanted due to the risk of portal vein thrombosis. In most patients a continuing deterioration of islet function is observed after transplantation.^1^ It is unsure whether intraportal transplantation is the preferred site to transplant novel beta cell sources such as stem cell islets.^5^ Preferably, such cells are better transplanted to a site where monitoring and retrieval of the graft are possible. These shortcomings of intraportal islet transplantation ask for the exploration of alternative transplantation sites.

Extrahepatic islet transplantation can be accommodated by using biomaterial-based devices for encapsulation. Preclinical data show that the use of these cell delivery devices improve function and survival of islet grafts in non-hepatic sites.^6-8^ However, convincing data in larger animal models which come closer to the clinical situation is scarce.

Several islet delivery devices (IDDs) have been studied that were implanted in the subcutaneous tissue, as this is generally considered an advantageous site in terms of surface area, ease of implantation, graft monitoring and graft retrievability. However, the subcutaneous site has proven challenging and should be optimized to improve oxygen tension, vascularization and prevent fibrous tissue formation in and around the device.^9-13^

To achieve this, we developed an IDD with micropores in the polyvinylidene fluoride membrane allowing vascularization throughout and retrievability for eventual replacement.

Importantly, to bridge the gap between preclinical models and clinical use it is important to acknowledge the difference between the preclinical laboratory situation and a clinical study. In the latter often separate cell manufacturing, cell loading technology and clinical/surgical teams exist, calling for meticulous planning and communication between such teams. Clinical use of cell therapy calls for stringent adherence to regulations and embedding into existing infrastructure. Therefore, we designed a study aiming to mimic a clinical scenario in which primary human islets are seeded into devices in a standardized reproducible manner at the islet isolation center, handled and shipped in sterile conditions in dedicated closed containers to the operating theatre, then handed over to the surgical team for subcutaneous implantation in a porcine model. In addition, sites prone to less or more friction due to muscle movements were compared after 3 months of implantation, based on engraftment and the local tissue reaction to the device. We included sites that were expected to have different degrees of movement to observe potential differences in inflammatory response due to friction which can lead to an enhanced foreign body response to the implanted device.

## Material and Methods

### Islet Delivery Device

Open microwell-array islet delivery devices were manufactured from polyvinylidene fluoride (PVDF). Devices had 400 μm wide microwells with a depth of 250 μm and pore diameters ranging between 30-90 μm. Polymer films used as lid held a pore pitch of 100 μm and pore diameter of 40 μm. Each device had a support ring. Each device held 3000 microwells distributed over an oval shape with dimensions of 27 × 44 mm.^14^ The devices were gamma sterilized. Further specifications can be found in Supplementary Methods.

### Animals

Six Topigs Norsvin TN70 pigs, all sows, were obtained from a commercial breeder (Van Beek SPF Varkens B.V., Lelystad, the Netherlands). The animal experiments were conducted at the Central Laboratory Animal Research Facility of the University Utrecht with approval of the Animal Welfare Bodies of the Leiden University Medical Center and University Utrecht. The pigs were housed in pairs, received standard animal facility chow (energy-reduced pig chow) twice daily, ad libitum water, and were weighed 1-2 times per week. The pen was enriched with a toy chain with chewing stick.

### Human Islet Isolation

Pancreatic islets were supplied by the Human Islet Isolation Laboratory of the Leiden University Medical Center. The pancreas donor was a 50-year old female who donated after circulatory death. The islets were isolated as previously reported and cultured overnight.^15^ A fraction of 90% purity was used to seed IDDs with 5.000 islet equivalents (IEQ) each.

### Seeding of Devices

An in-house designed stainless-steel clamping tool was used to fixate the devices and prevent islet loss during seeding and handling. A 50 mL syringe (BD Plastipak) fastened in a retort stand with burette clamps was connected to a 5 Fr blunt-tip feeding tube (Covidien Argyle 460802E) which was inserted into the seeding opening of the device. Devices were then clamped finger tight into the seeding tool with wing nuts. The feeding tube and device were flushed with medium to remove air. The devices designated to be co-implanted without human islets were treated in the same manner, except that instead of adding an aliquot of islets, islet-free medium was added to the syringe to sham-seed the device. Samples were taken from the islet medium to assess growth of microorganisms. Seeding of the devices was randomized and surgeons were blinded to the presence or absence of islets in the device.

### Transport of Devices

Devices seeded with islets and empty devices were treated in the same manner and transported in pairs in 500 mL medium-filled PMP wide-mouth containers (Nalgene) while keeping them fixed in the clamps. Both islet-seeded and empty devices were transported in medium with a pH of 7.2-7.4 containing Ringer Acetate, human albumin, glucose and CaCl_2_.The containers were packaged in sterile bags and transported to the Animal Research Facility. A schematic overview of islet seeding, transportation and implantation is depicted in Figure S1. Empty IDDs implanted in the last four pigs were brought to the facility in the packaging in which they were sterilized.

### Experimental Set-Up

The anterior neck was selected as a high-friction site, whereas the hind legs and abdominal subcutis were selected as low-friction sites. To implant the devices, a 5 cm incision was made parallel to the skin tension lines. Subcutaneous pockets were created by blunt and diathermic dissection on both longitudinal sides of the incision. A dummy device was first fitted to evaluate pocket size to ensure sufficient space was available for device insertion. The devices were then inserted with the microwells posteriorly. All IDDs were inserted 1 to 2 cm away from the incision to prevent suture overlay after wound closing. Sham sites were prepared exactly the same as a regular implantation with a device, with the exception that after fitting the site with a dummy, no device was inserted.

The subcutaneous pockets were closed separately with running sutures of Vicryl 3-0, followed by intracutaneous suturing of the skin with Monocryl 2-0. To remove all devices after 3 months, the same incision was used for retrieval of the grafts and directly surrounding tissue. The skin was subsequently closed with intracutaneous sutures. In total, 8 islet-seeded and 26 empty devices were implanted across 6 animals. Devices were implanted for three months. The experimental methodology is outlined in Table S1.

### Scoring Chart

Wound healing was monitored according to a scoring chart based on the clinically used ASEPSIS and Southampton scores, taking into account the typical characteristics of inflammation.^16,17^ The components of the score were necrosis, erythema (skin redness), divergence of the wound, pus, edema and overall illness of the animal. Wounds of the pigs implanted only with empty IDDs were scored daily by experienced animal caretakers in the first two weeks after the operation, followed by weekly scoring. The scoring chart can be found in Table S2.

### Histology

Devices were explanted after 3 months and immediately placed for 48-72 hours in 10% neutral buffered formalin (Sigma-Aldrich). Samples were then gradually dehydrated with an increasing ethanol concentration series ranging from 70% to 100%, immersed in xylene and embedded in paraffin for histology. Serial 8 micrometer thick tissue sections were stained with haematoxylin and eosin (HE), or Masson’s Trichrome staining (HT15, Sigma-Aldrich). Processed tissue sections were subsequently imaged using a Nikon Eclipse Ti inverted microscope equipped with a DS-Ri2 camera and CoolLED pE100 light source at 4× and 40×. Histological evaluation was done using FIJI software (https://fiji.sc/).

## Results

### Surgical Feasibility and Challenges in Islet Delivery Devices

In the first experiment, the complete clinical procedure of islet isolation, seeding, transportation and transplantation using an IDD was mimicked. These devices were loaded with human pancreatic islets using a dedicated cell seeding clamp and feeding catheter, ensuring homogenous dispersion of islets inside the device (data not shown). For 6 out of 8 devices, no islets were detected in drained medium, while less than 10% of seeded islets could be detected in the drained medium of the remaining 2 devices. Following this, sixteen devices were bilaterally implanted in the subcutaneous tissue of the anterior neck and on the lateral hip of two pigs using standard surgical tools. Each site received one seeded and one empty device without complications. An inflammatory response with swelling, erythema and/or non-healing wounds was observed in all four implant sites in both pigs, leading to 10 islet-seeded and empty devices being expelled. After 10 days, all remaining 6 devices were retrieved.

**Figure 1.**
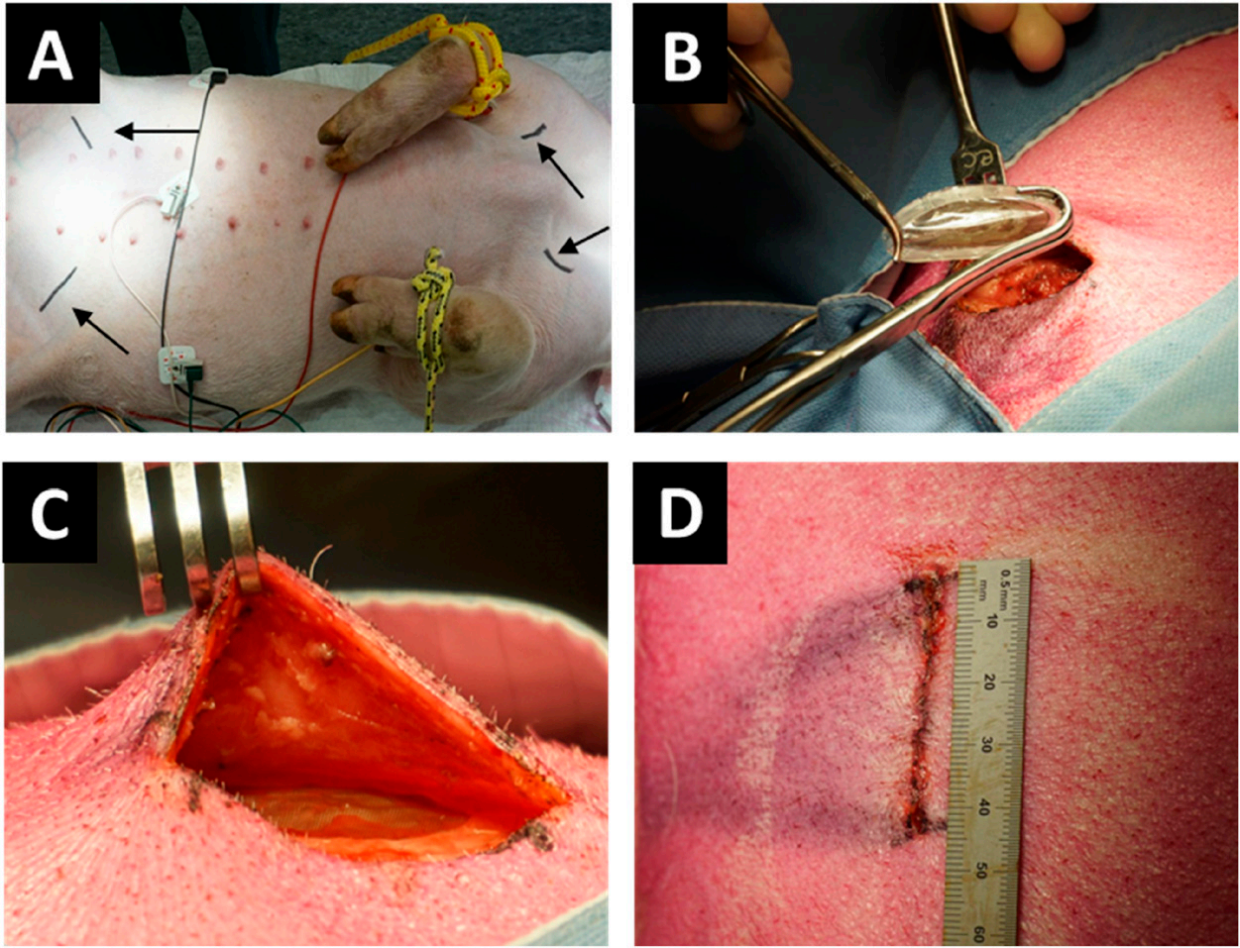
Implantation procedure. (A) Marking of the implantation sites for the abdomen and neck. Black arrows point to markings. (B) Fixation of the device with regular surgical equipment. (C) Device in situ. (D) Measurement of wound size.

To evaluate the long-term tissue response to the device in subcutaneous low and high-friction sites, a follow-up experiment was conducted using empty IDDs in 4 pigs. Signs of inflammation were scored using a combined scoring method. In two pigs, singular empty IDDs were bilaterally implanted in the subcutis of the anterior neck and lower abdomen, with one sham site per pig. Another pair of pigs received IDDs implanted in pairs in the same site. All devices were safely implanted without causing damage using regular surgical equipment. The mean (SD) time for implanting one IDD per site was 17.9 (3.9) minutes and 23.5 (5.2) minutes for two IDDs per site. The devices implanted at the abdominal site (Figure 1) remained palpable throughout the implantation period. After three months, all devices were retrieved without complications.

### Foreign Body Response at Different Implant Sites

**Figure 2.**
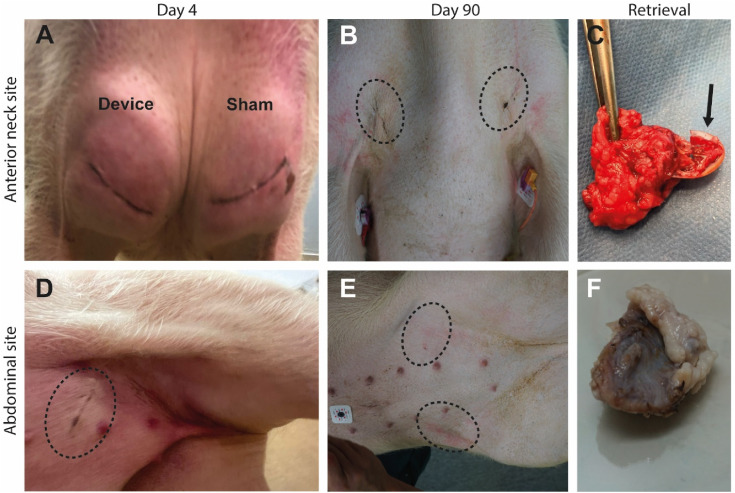
Tissue response and healing of wounds. Devices were implanted in the anterior neck (A–C) or abdominal site (D–F). Wound assessment four days after implantation (A, D) and three months after implantation (B, E). Retrieval of devices (C, F). The device retrieved from the neck displays a loss of macrostructure (black arrow). Location of transcutaneous incisions are marked with a dashed black oval in B, D and E.

**Figure 3.**
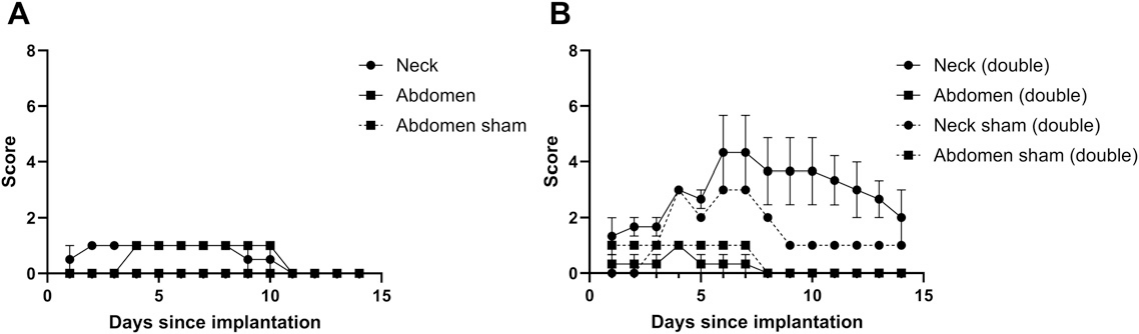
Wound scores for pigs implanted only with empty devices subcutaneously in the neck and abdomen. (A) wound scores for singularly implanted devices per site. (B) wound scores for devices implanted *in duplo* per site. A low score indicates a better outcome. Displayed in mean + SEM.

The inflammatory response was more pronounced in the neck and hind legs compared to the abdomen. This is illustrated in Figure 2A and D. The inflammatory response in the abdomen primarily involved minor edema, and subsequently regressed with a score of 1 at maximum that started on the first postoperative day. Daily wound scores indicated a higher severity (with a maximum score of 5 on day 6-7) in the anterior neck site compared to the abdomen, although the data size was insufficient for a robust conclusion (Figure 3). One pig with singular, empty IDDs per site succumbed to an unrelated undiagnosed cardiac condition on the day of implantation. A list of all adverse events is provided in Table S3.

**Figure 4.**
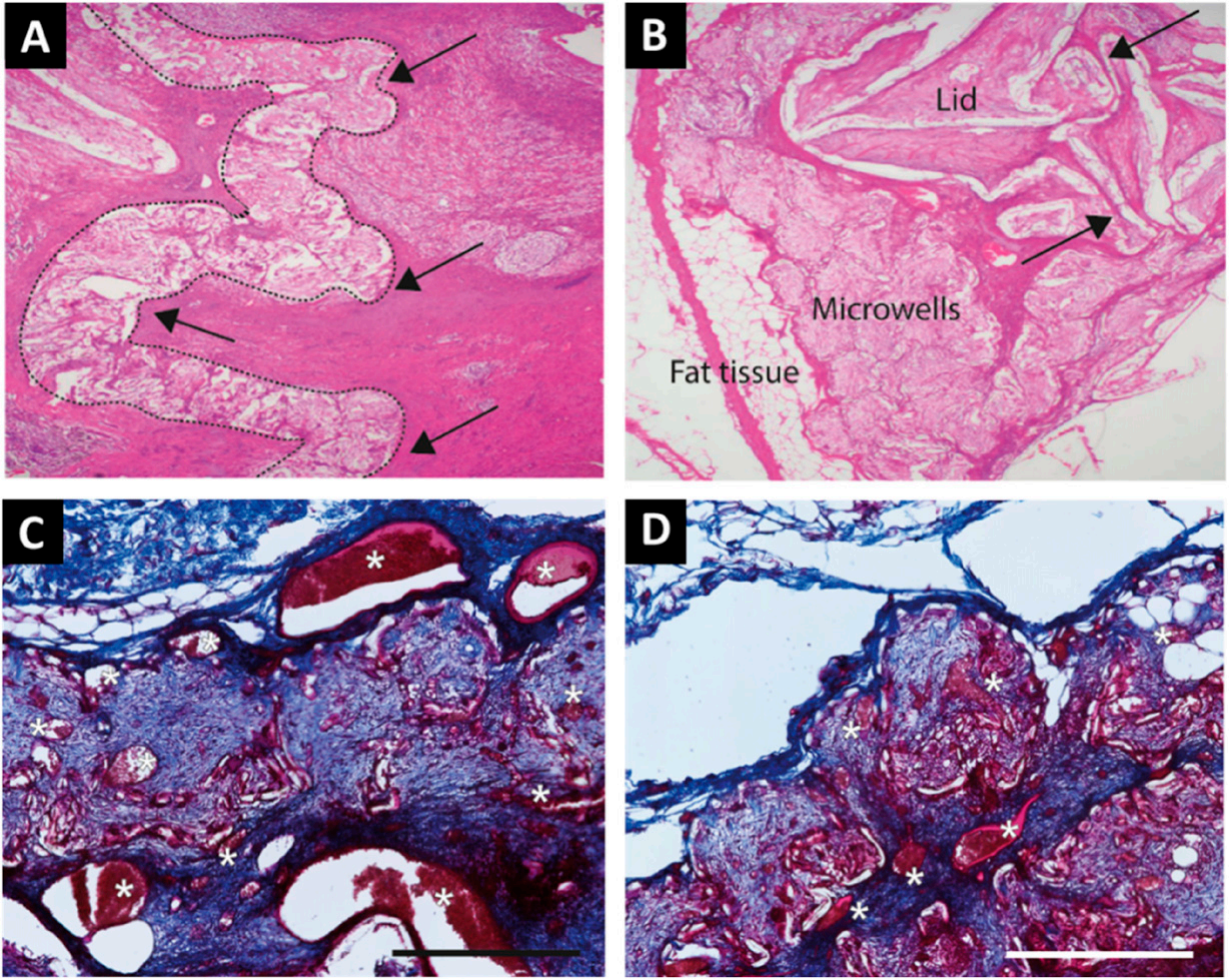
Tissue response to microwell devices. Tissue sections of microwell devices located in the neck (A) and abdomen (B) were stained with hematoxylin and eosin. The arrows indicate folding of the original flat structure of the device, and the microwell area is highlighted with a dashed line in A. (C, D) Two different tissue sections of the devices located in the abdomen stained with Masson’s trichrome staining with blood vessels in red (also marked with white *) and fibrous tissue (blue), imaged at 20× magnification, scale bar indicates 400 μm.

Nine empty IDDs underwent histological evaluation after three months. During explantation, two devices could not be retrieved due to unwitnessed loss in an earlier phase of the study, and four were excluded from histological evaluation due to improper fixation. The remaining devices exhibited a loss of their original flat macrostructure (Figure 2C, F and 4A, B), revealing multiple layers of microwells, indicative of device folding. Despite this, the individual subspace within microwells remained intact. Histological examination showed moderate to marked granulomatous inflammation of the pericapsular tissue, accompanied by adipose tissue degeneration. Qualitative analysis indicated mild general inflammation, and fibrovascular ingrowth comprised loosely-packed thin bands of well-vascularized connective tissue (Figure 4A and B). The tissue consisted of fibrovascular stroma which was typically characterized by fine (grade 1-2) bands of connective tissue and well-established vascular channels that were mostly small to moderately sized (grade 1-2) with occasional large vascular channels, as highlighted in the Masson’s trichrome staining (Figure 4D and E).

## Discussion

In this study, we mimicked a clinical islet transplantation using a dedicated open microwell islet delivery device. We specifically focused on the logistical procedure, surgical intervention and the feasibility of subcutaneous implantation and retrieval of IDDs at different sites in Landrace pigs. We demonstrated that these devices could be seeded with islets and implanted with regular surgical equipment without complications or damage to the device. This study is only one of the very many steps in bringing islet cell delivery devices to the clinic. Nevertheless, these steps are pivotal to finally bridge the gap between experimental success and clinical use.

High (neck) and low (abdomen, hind leg) mobility sites were selected to observe the effect of friction on inflammatory response and structural integrity of the implanted devices.^18^ Macrostructure of the device was more affected in high-friction sites. The observed folding of the devices, even in the abdominal site (although to a lesser extent), indicates that additional support and rigidity may be required to fully maintain the macrostructure of the device. However, excessive rigidity of the subcutaneous device may also be more mechanically and cosmetically burdensome for a recipient. In a large survey, patients with type 1 diabetes indicated that islet delivery devices should not hinder daily activities.^19^ It was suggested previously that subcutaneous implants should have a small width, an ergonomic shape with well-rounded angles and should be made of a smooth material.^20^ This is in line with our current design.

The individual subspace between microwells was well maintained in the low-friction site, that is the abdominal wall due to less tissue contraction and foreign body response. Despite having sufficiently large pockets to fit the devices measured by a dummy beforehand, there may have been more friction in the anterior neck than initially expected to occur at this site. Relative movement between the implant and the surrounding tissues may have led to mechanical disruption likely causing continuous exposure to microtraumas to the surrounding tissue. This in turn induced a more severe inflammatory response due to tissue damage preventing gradual healing leading to fibrous tissue formation.^21^ Histological analysis of our samples show that the devices were intact but lost their original form. An ongoing foreign body response was observed, which was partially attributed to the expected reaction to the device. This effect is surgery and site dependent since a similar tissue reaction was observed in the sham sites of the neck. Our observations indicate an inter-site variation in inflammatory response in pigs which has been described before.^22,23^ Distance between the device and incision location was not considered a risk factor for failure of successful implantation, as all devices were implanted at the same distance and none of the abdominal devices were expelled.

Tissue ingrowth was present in all examined devices without severe fibrous encapsulation. This can most likely be attributed to the choice of PVDF as an inert biomaterial and the design of the device.^24,25^ Appropriate oxygenation is a critical requirement of any therapy with pancreatic islets.^26^ Small and several large vascular channels were present in and around the microwells of abdominally implanted IDDs. The degree of vasculature ingrowth in a medical device was previously shown to be influenced by the pore size, with an optimal vascular ingrowth for pores between 30 - 50 μm.^27-31^ In our experiment, all assessed devices had ingrowth of vasculature. This is consistent with results from a previous study, in which a first-generation device with a similar design showed promising results in rodents.^14^

This study has several limitations. Human pancreatic islet survival and function could not be assessed as both islet-seeded and co-implanted empty devices were likely expelled due to a xenogeneic immune response in an immunocompetent pig model as the devices were transported in human albumin-containing medium and each site had a device with human islets.^26,32^ Furthermore, the model used has an inherent risk of wound dehiscence and device loss because of their subcutaneous location in sites sometimes susceptible to friction and rubbing by the pigs. Still, our study demonstrated the feasibility of all aspects of clinical translation of this IDD, from isolation to transportation, implantation and long-term retrieval without major hurdles. Outcomes of our study revealed that low-friction sites without frequent movements are preferred for subcutaneous implantation since there is a less foreign body reaction observed in these sites.

In conclusion, the complete process of handling, loading and surgically implanting (and retrieving) the PVDF microwell islet delivery devices is feasible. However, high mobility implant sites such as the neck should likely be avoided to achieve successful implantation. Design of the IDDs to maintain integrity during movement and the choice of a low mobility implant site are likely to be important factors for successful future beta cell replacement therapy using macroencapsulation devices.

## Supplemental Material

Supplemental Material - Subcutaneous Implantation of Open Microwell Islet Delivery Devices in Pigs Supplemental Material for Subcutaneous Implantation of Open Microwell Islet Delivery Devices in Pigs by Maarten C. Tol, MD, Rick H. W. de Vries, PhD, Marten A. Engelse, PhD, Françoise Carlotti, PhD, Aart A. van Apeldoorn, PhD, Eelco J. P. de Koning, MD, PhD, and Volkert A. L. Huurman, MD, PhD in Surgical Innovation

## Appendix

### Abbreviations

HE: haematoxylin and eosin
IBMIR: instant blood mediated immune response
IDD: Islet delivery device
IEQ: Islet equivalents
LUMC: Leiden University Medical Center
PVDF: polyvinylidene fluoride
