# Supplementary material for: Subcutaneous Implantation of Open Microwell Islet Delivery Devices in Pigs
Source: Surg Innov. 2024 Dec 13;32(2):141–8. doi: 10.1177/15533506241306491 (PMC11894865; doi:10.1177/15533506241306491)
Supplement: Supplemental Material - Subcutaneous Implantation of Open Microwell Islet Delivery Devices in Pigs [file sj-pdf-1-sri-10.1177_15533506241306491.pdf]

SDC, Materials and methods

Device manufacturing

Open microwell-array islet delivery devices (also indicated as open microwell devices) were manufactured as previously reported.[1] In short, a 15% (w/w) polyvinylidene fluoride (PVDF, Kynar 720, Solvay) in dimethyl formamide (DMF, Sigma-Aldrich) solution was casted onto a glass plate located on top of an automatic film caster (Elcometer) at 100 °C and 10% humidity. A universal applicator with gap distance of 250 µm was used to spread the polymer solution, and the created film was allowed to dry overnight at 100 °C under nitrogen gas flow. The resulting 15-µm thick films were subsequently washed overnight in endotoxin-free water and air-dried. The polymer films were made porous by laser micromachining with a UV short-pulse laser (Veld Laser Innovations B.V., the Netherlands). Polymer films used as lid held a pore pitch of 100 µm and pore diameter of 40 µm. Other films were reshaped into microwell-array films by means of microthermoforming, resulting in 400 µm wide microwells with a depth of 250 µm and pore diameters ranging between 30-90 µm. Thin polymer films were reinforced with a PVDF support ring aiming to prevent folding and provide surgical handling. The support ring was produced by loading PVDF pellets into a stainless-steel mold of 10 x 10 cm with a negative imprint of a disc with a diameter of 9 cm and thickness of 200 µm. The mold and encased PVDF pellets were loaded in a hydraulic press (Specac) and the pellets were molten into a 200 µm thick disc. The PVDF discs were subsequently cut to shape with a cutting plotter (Silhouette Cameo 4). The devices were assembled by ultrasonic welding of a support ring, microwell-array film and porous lid at specific locations with a manual LPS ultrasound welding station (Branson, the Netherlands) according to a custom made welding guide. Each devices holds 3000 microwells distributed over an oval shape with dimensions of 27 x 44 mm. The devices were incubated in ethanol, air-dried, washed in sterile endotoxin-free water and triple packaged in sterilization bags. Finally, the devices were gamma sterilized at 40 kGy by an ISO-certified commercial service (Steris Synergy Health).

Analgesia, anesthesia and antibiotic treatment

Pigs were fasted overnight prior to surgery. Premedication (ketamine 10mg/kg; midazolam 0.4mg/kg; atropine 0.05mg/kg) was administered intramuscularly prior to the procedure. Anesthesia was induced with thiopental (4 mg/kg) intravenously and maintained with midazolam (1mg/kg/hour) and sufentanil (10 mcg/kg/hour) intravenously. Meloxicam (0.4mg/kg) was administered intravenously prior to incision. Amoxicillin/clavulanic acid (10 mg/kg) was administered intravenously prior to incision and twice orally on the first postoperative day.

Table S1. Tabular overview of implantation and retrieval of islet delivery devices.						
	**Pig 1°**	**Pig 2¶**	**Pig 3**	**Pig 4†**	**Pig 5**	**Pig 6**
Neck						
Islet-seeded IDDs	2/1	2/0	-	-	-	-
Empty IDDs	2/1	2/1	2/2	1/NA	4/2§	2/0ǂ
Sham implant IDDs	-	-	0/NA	1/NA	0/NA	2/NA
Time until planned retrieval	3m	3m	3m	3m	3m	3m
Time until actual retrieval	10d	10d	3m	N/A	3m	3m
Hind leg						
Islet-seeded IDDs	2/1	2/0	-	-	-	-
Empty IDDs	2/2	2/0	-	-	-	-
Sham implant IDDs	-	-	-	-	-	-
Time until planned retrieval	3m	3m	NA	NA	NA	NA
Time until actual retrieval	10d	10d	NA	NA	NA	NA
Abdomen						
Islet-seeded IDDs	-	-	-	-	-	-
Empty IDDs	-	-	1/1	2/NA	2/2	4/4
Sham implant IDDs	-	-	1/NA	0/NA	2/NA	0/NA
Time until planned retrieval	NA	NA	3m	3m	3m	3m
Time until actual retrieval	NA	NA	3m	NA	3m	3m

Data shown as number of IDDs (islet delivery devices) implanted / retrieved during surgery. Time shown in days (d) or months (m). NA: not applicable.
° Pig 1: all non-retrieved IDDs were retrieved from the pen.
¶ Pig 2: all non-retrieved IDDs were retrieved from the pen.
†Pig 4: succumbed to an unrelated undiagnosed cardiac condition on the day of implantation.
§ Pig 5: 2 IDDs from the left side of the neck were not found during the retrieval surgery. No IDDs were observed to be lost.
ǂPig 6: it was observed that one empty IDD from the neck was lost in the early post-operative period. The other was not found during the retrieval surgery.

Table S2. Wound scoring chart

	**Erythema**	**Pus**	**Deviation**	**Necrosis**	**Edema**	**Overall sickness**
**0**	No erythema	No pus	No deviation	No necrosis	No edema	Animal is not sick
**1**	Mild erythema around wound	Moderate amount of pus	Deviation <0.5 cm	Visible or scented necrosis	Trace of edema	Animal is sick.
**2**	Fiery erythema <1cm from wound	Large amount of pus	Deviation <1 cm		Significant edema	
**3**	Fiery erythema <3cm from wound		Deviation >1 cm			
**4**	Fiery erythema >3cm from wound		Device is visible			

Each category to be filled out per implant site per animal.

**Table S3. Adverse events**		
Adverse event	Pigs:	Comment
Wound inflammation	1 and 2	All wounds showed erythema and edema from postoperative day 1 onwards. Both pigs were treated with antibiotics.
	3	Minor edema in the neck.
	5 and 6	Inflamed wounds in the neck: major edema and erythema, also present in the sham site. Both pigs were treated with antibiotics.
Wound deviation	1 and 2	All wounds were deviating progressively from postoperative day 2 onwards.
	6	A wound in the neck deviated on postoperative day 8 and healed *per secundam*.
Non-retrieved device	1 and 2	Six devices were retrieved during surgery on postoperative day 10, three devices were found in the pen. The remaining seven had been expelled but were not found.
	5	Two devices from the same site in the neck could not be found, despite the wound not having deviated in the postoperative course.
	6	One device in the neck was already expelled on postoperative day 9. The other device from the same wound could not be found and is presumed to be also expelled around that same day.

Pseudomonas fluorescens infected pancreatic islets	1 and 2	The culture medium of the pancreatic islets was contaminated with P. fluorescens. Cultures from the transport medium and swabs from the wound after retrieval were negative for this bacteria.
Possible slight constriction of thorax	6	A possible slight constriction of the thorax was noted with a minor impairment of hind leg strength. This was deemed unrelated to the experiment and did not cause any discomfort to the animal.
Death	4	This pig was found dead in the pen two hours after implantation. The cause of death was analyzed by root cause analysis. A pre-existing cardiac condition unrelated to the procedure was considered most likely.

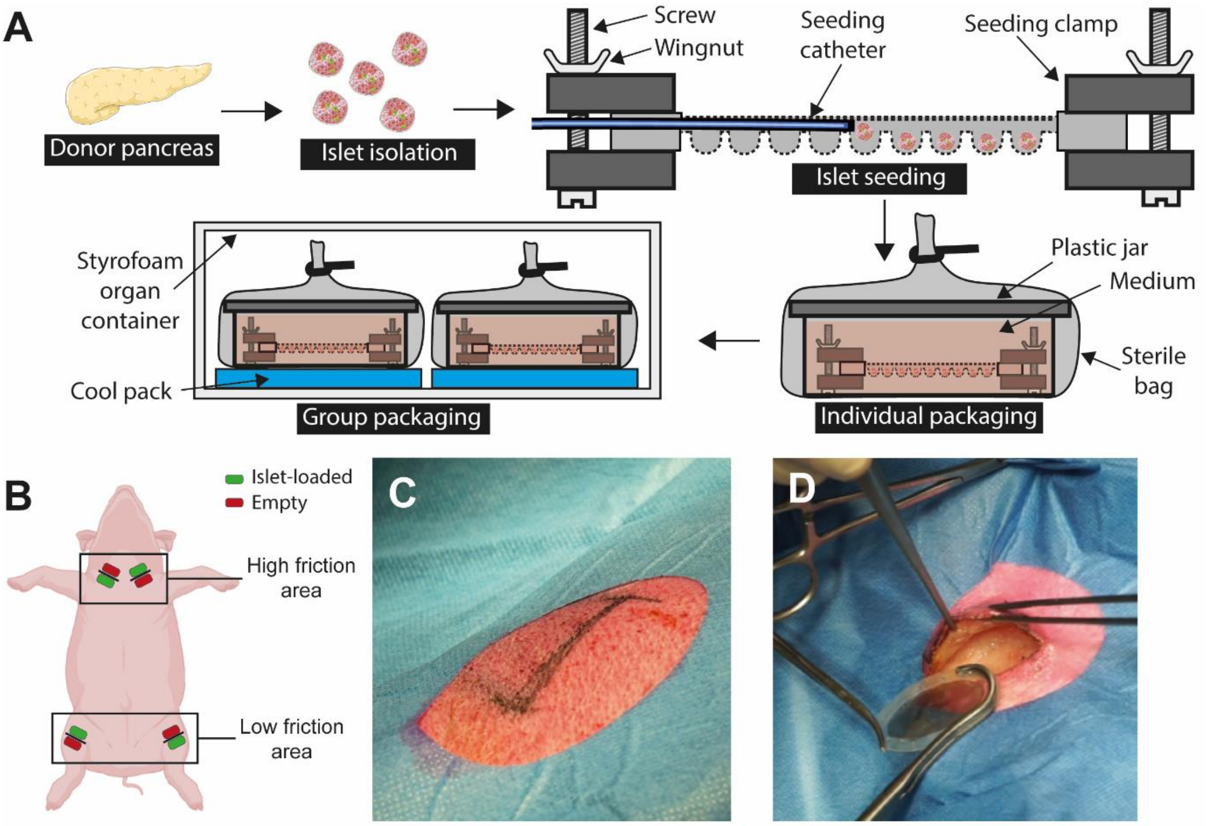

Figure S1: Implantation of islet-loaded microwell devices A) Overview of the steps required for seeding islets within the microwell-array islet delivery devices and subsequent transport to the operating theater. Devices are clamped in a seeding clamp to prevent leakage of islets out of the microwells during seeding. Devices and clamps are then packaged in a plastic jar, which is subsequently filled with medium. Finally, the jar is packaged in a sterile bag, and collected into a Styrofoam organ container filled with cool packs and transported on a cart. B) Implantation strategy of empty and islet-loaded devices C) Implantation site covered with sterile surgical drapes. D) Implantation of a sentinel device in the hind leg using a Cooley clamp covering the curvature of the device and Mosquito clamp covering the seeding inlet of the device.

REFERENCE LIST

1. Buitinga M, Assen F, Hanegraaf M, et al. Micro-fabricated scaffolds lead to efficient remission of diabetes in mice. *Biomaterials*. Aug 2017;135:10-22. doi:10.1016/j.biomaterials.2017.03.031
